# Power is not performance: Analysis of laser‐use metrics from a randomised clinical trial comparing low‐ and high‐power ureteroscopic lithotripsy

**DOI:** 10.1002/bco2.70182

**Published:** 2026-03-31

**Authors:** Mathias S. Æsøy, Christian Beisland, Øyvind Ulvik

**Affiliations:** ^1^ Helse Bergen HF, Department of Urology Haukeland University Hospital Bergen Norway; ^2^ Department of Clinical Medicine (K1) University of Bergen Bergen Norway

**Keywords:** active laser time, high‐power, laser lithotripsy, laser operating time, laser settings, low‐power, patterns of laser activation, renal stones, thulium fibre laser, ureteroscopy

## Abstract

**Objective:**

Thulium fibre laser (TFL) offers a wide range of power settings during ureteroscopic (URS) lithotripsy, yet the impact of power selection on intraoperative laser‐use dynamics remains unclear. The objective of this study was to characterise differences in laser activation patterns between low‐power (LP) and high‐power (HP) TFL during sheathless URS.

**Materials and Methods:**

This was a predefined secondary analysis of intraoperative laser data collected during a randomised clinical trial comparing LP (0.4–0.6 J × 10 Hz; 4–6 W) and HP (0.4–0.8 J × 20–40 Hz; 16–18 W) TFL URS lithotripsy. Flexible URS was performed without a ureteral access sheath. Laser system logs provided time‐stamped activation and pause events with microsecond precision. Laser‐use dynamics were characterised using a predefined set of complementary metrics, including active laser time (ALT), laser operating time (LOT), total activation count, activation durations, pause durations and operator duty cycle (ODC = ALT/LOT). Group comparisons used Holm–Bonferroni–adjusted *p* values.

**Results:**

Data from 72 LP and 74 HP procedures were analysed. ALT was longer with LP (23 vs. 13 min; *p* < 0.001), whereas LOT was similar (31 vs. 30 min; *p* = 0.194). LP had a lower activation count (55 vs. 89; *p* < 0.001) and longer activations (median 24 s vs. 7 s; *p* < 0.001). Empirical cumulative distribution function analysis showed 89.4% of LP activations lasted ≤30 s compared with 97.2% for HP (*p* < 0.001). Total pause time was shorter in LP (8 vs. 14 min; *p* < 0.001), resulting in a higher ODC (73% vs. 49%; *p* < 0.001).

**Conclusion:**

LP and HP TFL lithotripsy result in distinctly different laser‐use dynamics during sheathless URS. LP supported sustained, continuous lasing, whereas HP yielded fragmented activation requiring frequent pauses. Coupled with superior stone‐free outcomes in the parent trial, these findings suggest that preserving clear endoscopic visibility and more active lasing may be more impactful than increasing laser power.

## 1 INTRODUCTION

Thulium fibre laser (TFL) technology has rapidly emerged as a preferred energy source for ureteroscopic (URS) lithotripsy.^1^, ^2^, ^3^ TFL emits near‐infrared light at a wavelength (1940 nm) close to the water absorption peak, offering theoretical advantages such as a lower threshold of stone ablation, reduced retropulsion and improved efficiency compared with Holmium:Yttrium–Aluminium–Garnet (Ho:YAG).^4^, ^5^ Moreover, TFL offers a broader range of pulse frequencies (hertz [Hz]) and pulse energies (joule [J]), operating at power up to 60 W.^6^ This versatility introduces wide variability in clinical practice, especially regarding the preferred power range for safe and efficient TFL lithotripsy.^7^

High‐power (HP) settings have been promoted for faster stone ablation,^8^, ^9^, ^10^ while low‐power (LP) settings are thought to enhance safety by minimising heat generation and preserving endoscopic visibility.^11^, ^12^ Yet evidence from randomised clinical trials (RCT) indicates that increased power does not necessarily translate into improved outcomes.^13^, ^14^ In a recent RCT, we found that LP and HP TFL achieved comparable operative times, while LP improved stone‐free rates (SFR) significantly and reduced minor postoperative morbidity (Clavien–Dindo ≤2).^15^ These findings raise a key question: Do the advantages for LP reflect the power settings' capabilities of stone ablation, or differences in laser‐use dynamics between the LP and HP groups?

Despite growing adoption of TFL, objective descriptions on intraoperative laser activation patterns are sparse. Metrics such as activation frequency, activation durations, pause durations and operator duty cycle (ODC) may provide valuable insights into the ergonomics of TFL lithotripsy. These metrics capture the temporal structure of laser energy delivery in real time and may help reconcile discordant observations (e.g., faster ablation without shorter procedures). Understanding these parameters may be essential for interpreting efficiency and safety outcomes observed in clinical trials and may guide more standardised power recommendation for TFL use in everyday practice.

The present study represents a predefined secondary analysis of intraoperative TFL usage data from an RCT comparing LP and HP TFL URS lithotripsy.^15^ The aim was to characterise and compare laser‐use dynamics between groups, thereby enhancing our understanding of how power selection is associated with real‐time laser application, and to contextualise observed outcomes regarding efficiency, endoscopic visibility and SFR.

## 2 MATERIALS AND METHODS

### 2.1 Study design, setting and populations

This study is a prespecified secondary analysis of intraoperative laser usage data collected during an RCT comparing LP and HP TFL URS lithotripsy.^15^ The parent trial was conducted at Haukeland University Hospital in Bergen, Norway. Randomisation was performed 1:1 using computer‐generated blocks with concealed allocation. Inclusion and exclusion criteria have been described previously.^15^

Between 1 February 2023 and 22 May 2025, 150 day‐case URS procedures for renal stones 8–25 mm were randomised to LP (0.4–0.6 J × 10 Hz; 4–6 W) or HP (0.4 J × 40 Hz, 0.6 J × 30 Hz, 0.8 J × 20 Hz; 16–18 W). Power settings of 16–18 W were selected to maintain thermal safety during sheathless URS. Given that power levels above 20 W have been associated with hazardous intrarenal temperatures, 16–18 W was considered high‐power in this clinical context.^11^

### 2.2 Operative technique and data collection

Procedures were performed using a flexible digital ureteroscope (URF‐V3 or P7; Olympus, Tokyo, Japan) and a 150‐μm core TFL fibre (Soltive Premium 60 W; Olympus, USA). A dusting strategy was used in all cases. Stone material was collected for analysis by aspiration of dust through the endoscope's working channel. No basket extraction or fragment retrieval was performed. Irrigation was delivered via gravity (60 cm bag height), with no pressurised pump or suction system. No ureteral access sheath (UAS) was used. Baseline stone characteristics (size, volume, density, location and composition) and surgeon experience (resident vs. endourologist/consultant) were recorded prospectively in the parent RCT and were comparable between treatment arms, indicating similar procedural complexity.^15^

Upon completion of each case, laser data were extracted directly from the TFL system's internal memory. Each log contained timestamped activation and deactivation events with microsecond precision, as well as pulse energy, frequency and total energy delivered.

The following laser parameters were derived from each log: total number of activations, activation duration, longest activation, total active laser time (ALT), laser operating time (LOT, interval from first activation to last deactivation), operator duty cycle (ODC = ALT/LOT), pause durations, longest pause, total pause time and energy used. ODC is used as a time‐based descriptor of workflow continuity during the lasing phase (LOT). As all procedures were performed using a dusting‐only strategy, nonlasing intervals primarily reflect repositioning, visual clearing or re‐targeting rather than active stone treatment. Collectively, these metrics characterise laser‐use dynamics by describing the temporal pattern of laser application over the entire case.

## 3 OUTCOME

Predefined outcomes were laser‐use dynamics metrics derived from the system logs, including ALT, LOT, total number of activations, activation durations, pause durations, ODC and total energy delivered.

Exploratory outcomes: surgeon‐rated endoscopic visibility scores (5‐point Likert scale), collected prospectively in the parent randomised trial,^15^ were linked at the procedure level to laser‐use metrics derived from the system logs. Associations between visibility and selected laser‐use parameters were assessed using Spearman's rank correlation. These analyses were considered exploratory and hypothesis‐generating.

## 4 STATISTICS AND ETHICAL APPROVAL

Continuous variables were compared using independent‐sample *t* tests when normally distributed and the Mann–Whitney *U* test when distributions were nonnormal. Pearson's *χ* ^2^ test was used to compare categorical variables. All tests were two‐sided. Statistical analyses were performed using IBM SPSS Statistics 29.0.0.2. Given the secondary‐analysis nature of this study, multiplicity adjustments were performed using Holm–Bonferroni (familywise *α* = 0.05). Adjusted *p* values are reported. Statistical significance was set at *p* < 0.05. Python V3.12.4 with pandas (2.2.2) and matplotlib (3.9.2) libraries were used for visualisation.

Individual laser activation events are nested within procedures and therefore not statistically independent. Accordingly, the procedure was treated as the unit of analysis for inferential statistics, using case‐level summary metrics (e.g., ALT, LOT, total activations, activation durations and ODC). Figures are presented for descriptive illustration of laser‐use, representing workflow during the lasing phase (LOT), and were not used as the basis for independent‐sample hypothesis testing.

The study was registered on ClinicalTrials.gov (NCT05697250) and in the hospital's database for science (eProtocol ID‐3755) and was approved by the Norwegian Regional Ethical Committee (REC ID‐550740). Informed consent including a signed form was obtained from each participant.

## 5 RESULTS

TFL data logs were evaluated for each case included in the RCT analysis (73 LP and 75 HP). One incomplete dataset was identified in each group, resulting in 72 LP and 74 HP cases available for analysis of laser usage metrics.

**FIGURE 1:**
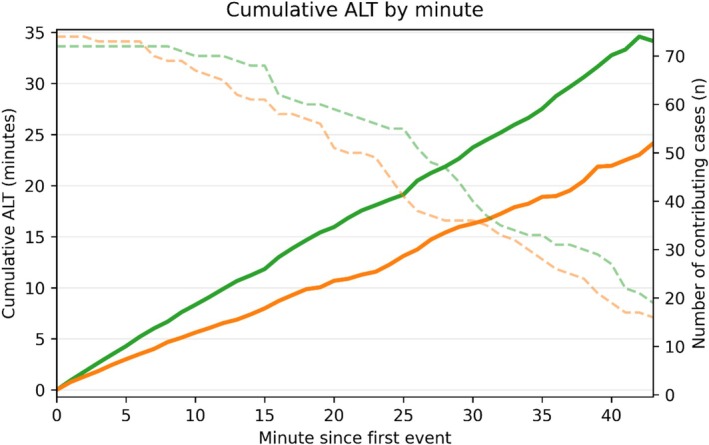
Cumulative active laser time (ALT). Solid curves show the median cumulative ALT from the first laser activation for each group (green = low power and orange = high power). A steeper slope indicates a higher proportion of time spent actively lasing relative to pauses. The dashed curves (right *y*‐axis) indicate the number of cases still contributing (*n*) at each minute, reflecting case completion over time. Values were calculated only while ≥15 procedures remained in each group, and the curves are cut beyond this point to avoid instability from small sample sizes.

Laser usage metrics are summarised in Table 1. ALT was significantly longer in the LP group compared with the HP group (23 min vs. 13 min, *p* < 0.001). LOT was similar between groups (31 min vs. 30 min, *p* = 0.194). Figure 1 illustrates cumulative ALT‐time per minute, showing that LP cases accumulated laser activity at a consistently higher rate throughout the procedures.

**TABLE 1:** Laser usage metrics.

Laser usage metrics	High‐power	Low‐power	*p* value^a^
Active laser time, median (IQR), min^b^	13 (7–19)	23 (13–38)	<0.001
Laser operating time, median (IQR), min^c^	30 (19–42)	31 (25–44)	0.194
Operator duty cycle, median (IQR), %^d^	49 (36–63)	73 (61–86)	<0.001
Laser activations, median (IQR), *n*	89 (55–236)	55 (19–104)	<0.001
Activation duration, median (IQR), s	7 (4–11)	24 (11–56)	<0.001
Longest activation, median (IQR), s	56 (29–85)	214 (91–472)	<0.001
Pause duration, median (IQR), s	7 (4–12)	7 (4–11)	0.754
Longest pause, median (IQR), s	109 (69–188)	81 (37–134)	0.008
Total pause time, median (IQR), min	14 (8–20)	8 (4–13)	<0.001
Energy used, median [IQR], kJ	12 (6–18)	7 (4–10)	<0.001

^a^ *p* values adjusted according to the Holm–Bonferroni method.

^b^ Cumulative time the laser is actually firing (‘pedal‐down’ time), as recorded by the console; pauses are excluded.

^c^ Time from the first laser activation to the last laser deactivation; includes all intervening inactive intervals.

^d^ Operator duty cycle is defined as total active laser time/laser operating time.

**FIGURE 2:**
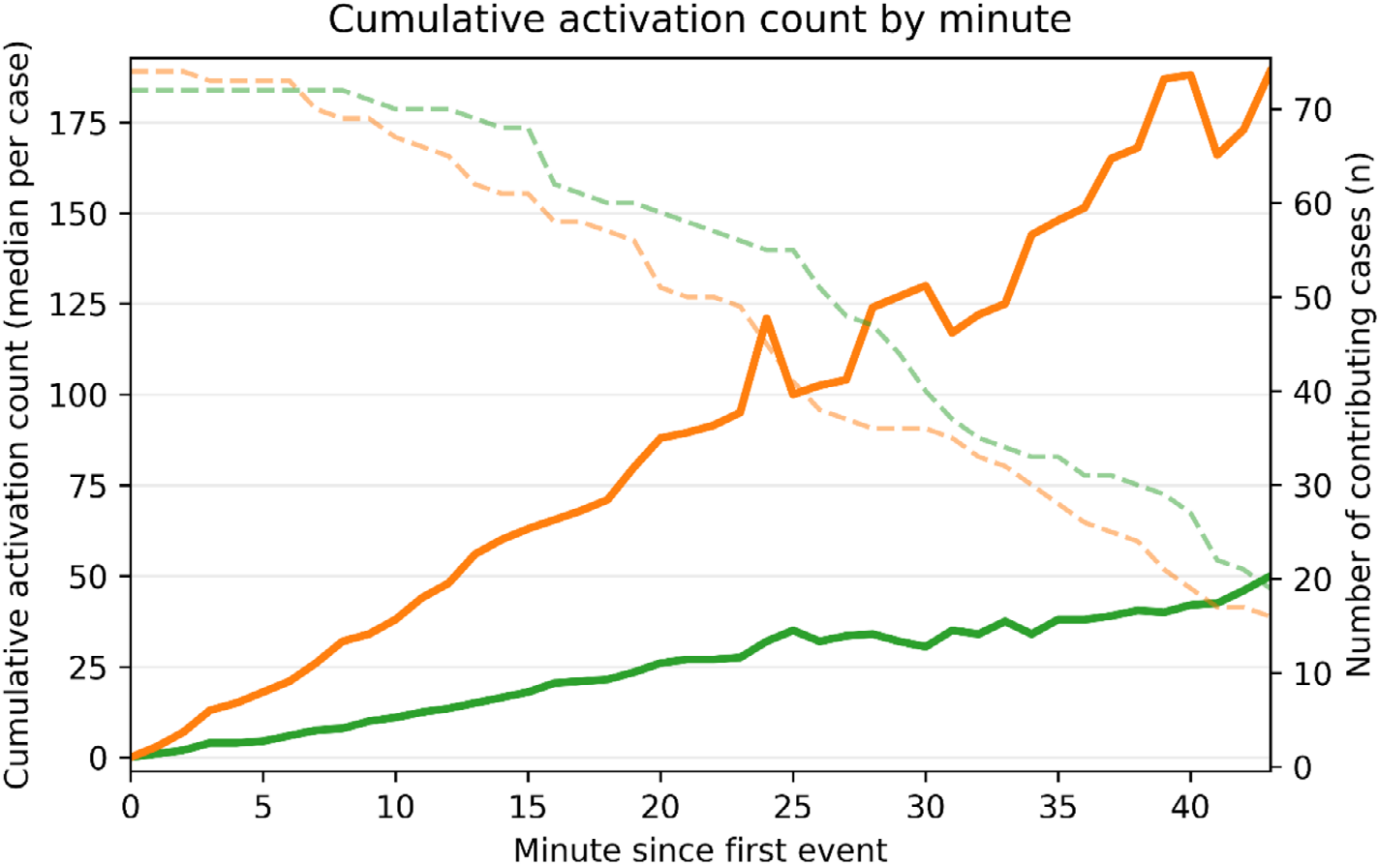
Cumulative laser activation count. Solid lines show the median cumulative number of laser activation events from the first laser activation (green = low power; orange = high power). A steeper slope reflects more frequent activation events during the procedure. Dashed lines (right *y*‐axis) indicate the number of cases still contributing data at each minute. Values were calculated only while ≥15 procedures remained in each group.

The total number of laser activations was lower using LP (55) compared with HP (89), *p* < 0.001. Figure 2 displays the cumulative activation count over time, demonstrating that HP cases accumulated laser activations at a faster rate than LP.

**FIGURE 3:**
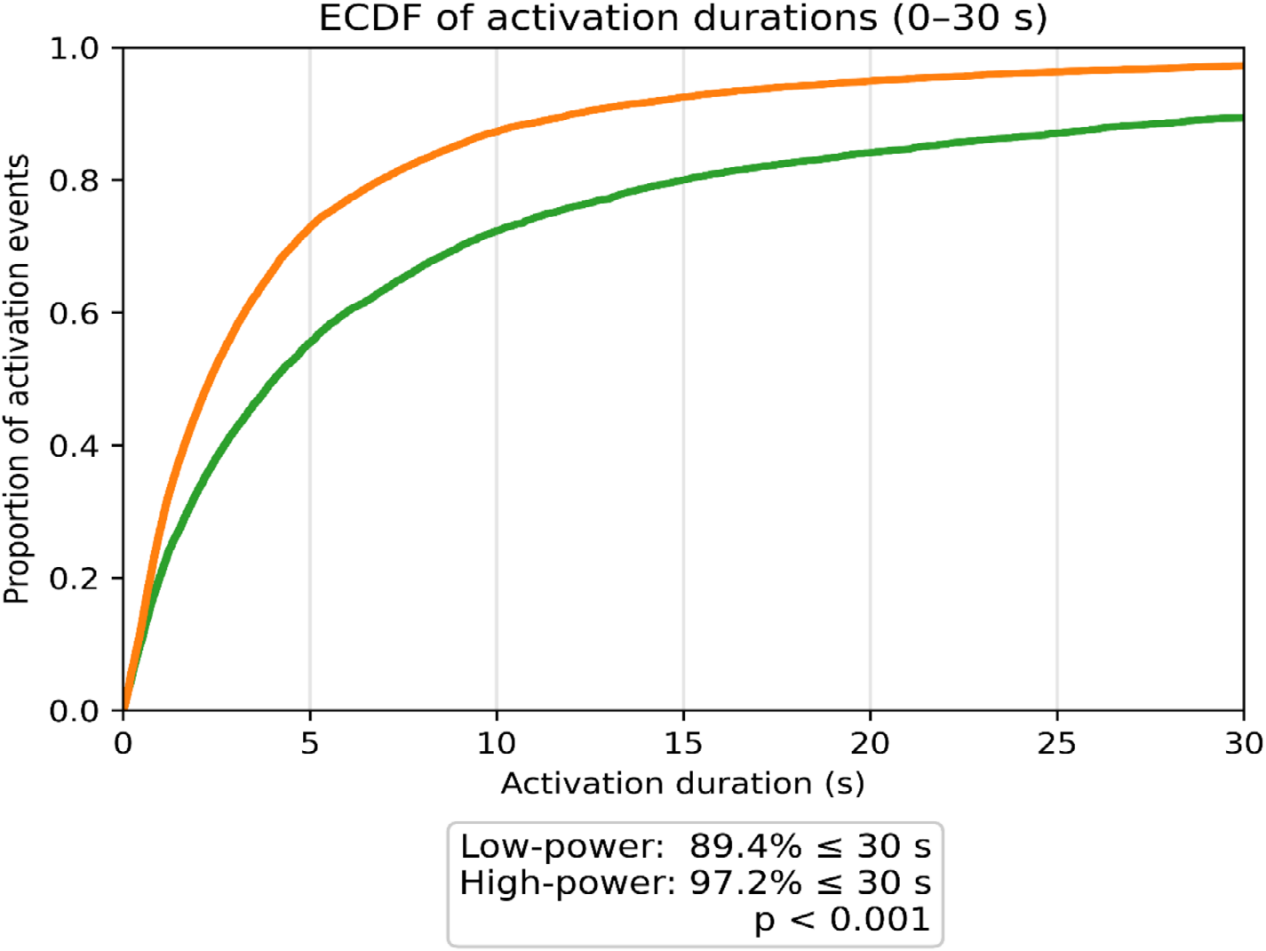
Distribution of laser activation durations (ECDF). Solid curves show the empirical cumulative distribution function (ECDF) of individual laser activation durations (green = low power; orange = high power). The *x*‐axis represents the duration of each activation event, and the *y*‐axis shows the proportion of activation events lasting up to that duration. The plot is displayed from 0 to 30 s, which covers the majority of activation events in both groups. Curve position reflects activation pattern: a left‐shifted curve indicates more short activations, while a right‐shifted curve indicates longer, more continuous activations.

Laser activation durations differed between groups. Median activation duration was longer with LP (LP 24 s vs. HP 7 s, *p* < 0.001). Furthermore, the longest activation duration (median) was 214 s in the LP group compared with 56 s in the HP group, *p* < 0.001. Figure 3 illustrates the empirical cumulative distribution function (ECDF) of laser activation durations (0–30 s). The ECDFs show a left shift for HP, illustrating a greater proportion of short activation bursts. Moreover, 97.2% of HP activations lasted ≤30 s compared with 89.4% of LP activations, *p* < 0.001.

**FIGURE 4:**
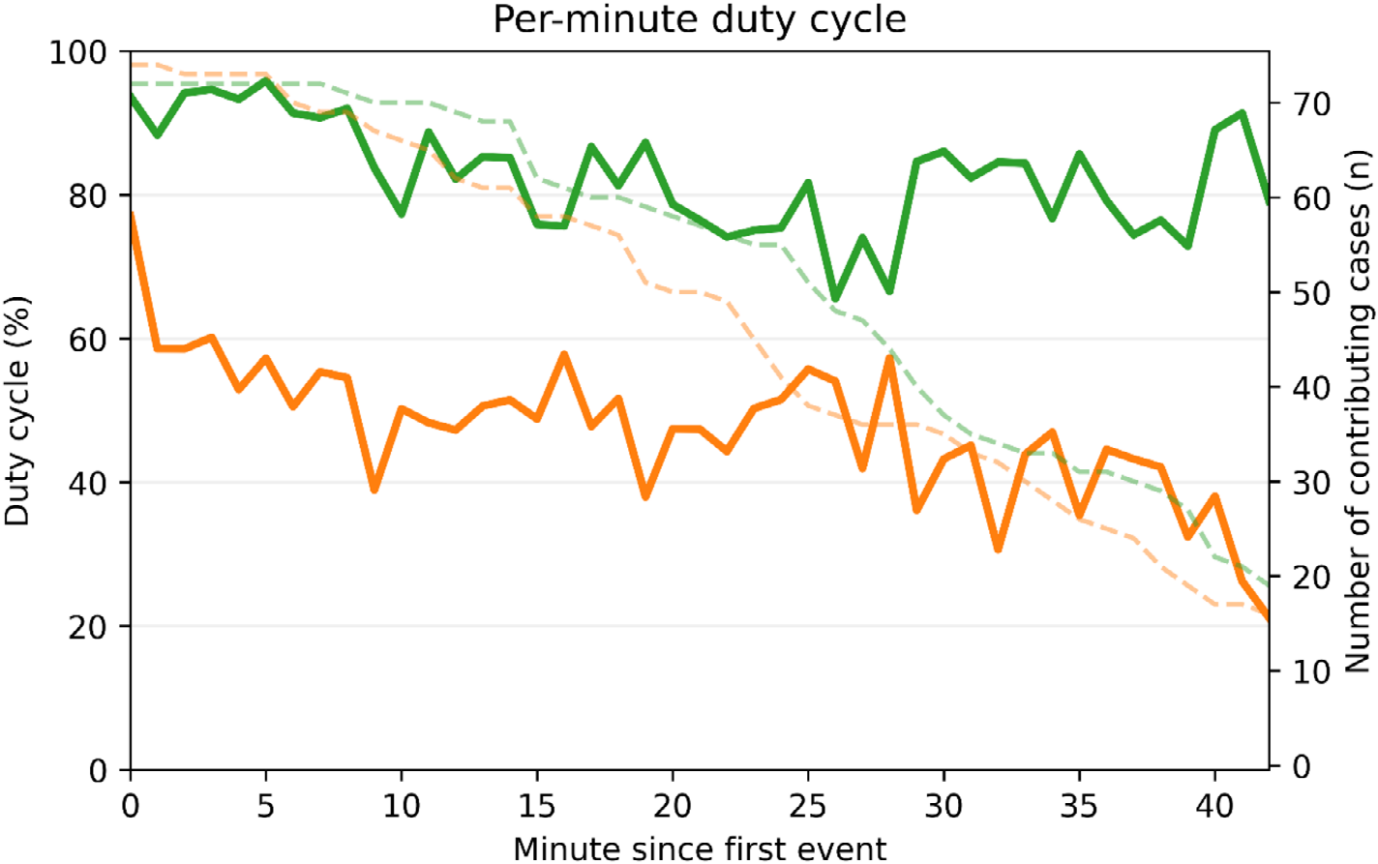
Operator duty cycle (ODC) by minutes since first activation. Solid lines show the median operator duty cycle (ODC) at each elapsed minute from the first laser activation (green = low power; orange = high power). ODC is defined as active laser time divided by laser operating time (ALT/LOT), representing the proportion of time spent actively lasing. A higher ODC reflects more continuous lasing with fewer pauses, whereas a lower ODC indicates a more stop–start pattern. Dashed lines (right *y*‐axis) indicate the number of cases still contributing data at each minute. Values were calculated only while ≥15 procedures remained in each group.

The median duration of pauses interrupting active laser periods was comparable between groups (Table 1). However, because HP cases had more frequent pauses, total pause time was longer compared with LP (HP 14 min vs. LP 8 min, *p* < 0.001). This difference is also reflected in the ODC; HP ODC was 49% compared with LP ODC 73%, *p* < 0.001. Figure 4 illustrates the per‐minute evolution of ODC throughout the procedure, with LP maintaining consistently higher values than HP at all time points. In the HP group, ODC progressively declined over time, whereas the LP curve remained comparatively stable.

**FIGURE 5:**
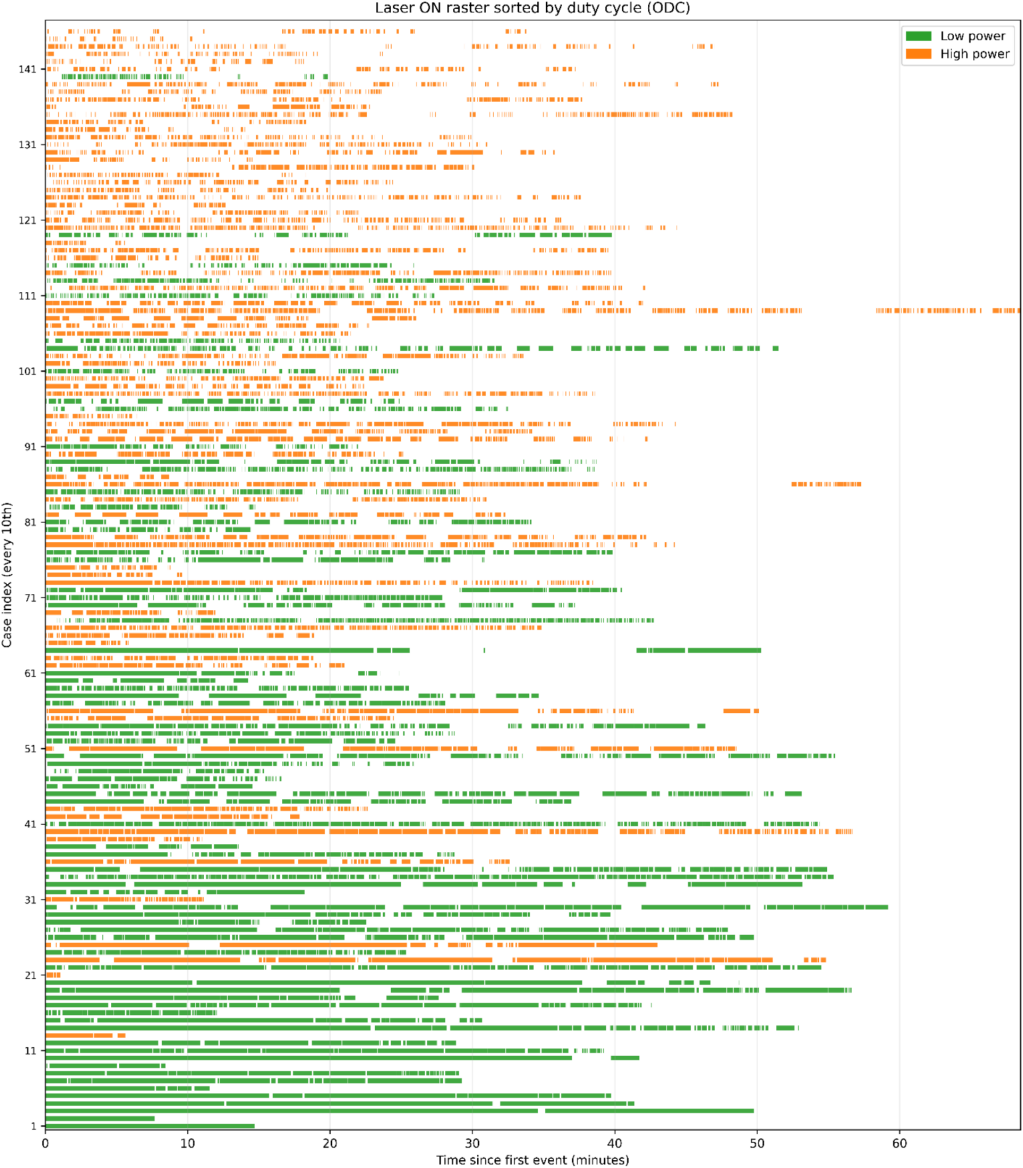
Temporal structure of laser activation events across all procedures. Each row represents a single URS procedure, ordered by ODC from highest (bottom) to lowest (top). Coloured bars denote individual laser activation events (green = low power, orange = high power), and bar length corresponds to activation duration. Low‐power procedures show longer, continuous activation segments with fewer interruptions, while high‐power procedures show short, frequent activation bursts separated by pauses.

Figure 5 depicts a raster plot of all procedures, ordered by ODC from high (bottom) to low (top). Each row is one case; coloured bars denote individual laser activations and their lengths (green = LP, orange = HP). Across the raster, LP cases display longer continuous activations segments, whereas HP cases show more frequent and short segments. Laser activation density is highest in the early minutes of the procedures and tapers of later.

Surgeon‐rated visibility scores showed a positive association with operator duty cycle (Spearman's *ρ* = 0.49, *p* < 0.001), mean activation duration (*ρ* = 0.42, *p* < 0.001) and longest activation duration (*ρ* = 0.46, *p* < 0.001) and a negative association with total number of activations (*ρ* = −0.36, *p* < 0.001). Higher visibility ratings were associated with more sustained laser activation, longer continuous lasing periods and fewer stop–start cycles.

## 6 DISCUSSION

This in‐depth laser analysis of our recent RCT demonstrates distinct differences in laser‐use dynamics between power settings, extending beyond differences in ablation rate alone. Despite shorter ALT with HP, LOT was comparable, and ODC favoured LP. In other words, how the laser is used, its activation pattern and continuity, emerge as a potential determinant of efficiency and outcome alongside the power settings intrinsic ablation capacity.

The laser activation dynamics diverged sharply according to power settings. LP produced a faster rise in cumulative ALT, indicating more sustained and continuous lasing (Figure 1). In contrast, HP accumulated ALT more slowly, consistent with more interruptions. Accordingly, HP accrued activations at a higher rate (Figure 2), reflecting short, stop‐start bursts rather than continuous runs. The ECDF confirmed predominantly brief activations with HP (97.2% ≤ 30 s vs. 89.4% with LP, *p* < 0.001; Figure 3). Finally, the raster plot demonstrates these patterns across all cases: Long and dense activation bands with LP are contrasted by short and discontinuous segments with HP (Figure 5).

The intermittent start–stop activation pattern observed in the HP arm may reflect adaptive surgeon responses to several effects associated with higher laser power. First, stone retropulsion may influence patterns of laser activation. However, although higher pulse energy can increase retropulsion,^16^ pulse energies were harmonised between arms except for an HP‐only 0.8 J (20 Hz) pop‐dusting setting. When pop‐dusting, typically towards the end of procedures, retropulsion is intended. Thus, a difference in retropulsion is unlikely to explain the differences in laser‐use dynamics, although retropulsion was not directly quantified in this study. Second, as HP lithotripsy increases thermal load compared with LP,^11^, ^17^ surgeons may have used the laser more cautiously with HP. However, HP was deliberately capped at 16–18 W to reduce thermal load during sheathless URS.^11^ Nevertheless, thermal safety considerations may have contributed to the intermittent laser activation pattern observed using HP.

Visibility constraints provide an additional mechanism through which surgeons may adapt their activation behaviour. Notably, in the parent RCT, surgeon‐reported endoscopic visibility was worse with HP (Likert 2.8) compared with LP (Likert 4.1), *p* < 0.001.^15^ In exploratory analyses linking laser‐use metrics with surgeon‐rated endoscopic visibility, higher visibility scores were associated with higher ODC, longer median and longest activation durations and fewer total activations. This pattern supports the hypothesis that visual clarity may be an important determinant of workflow continuity during sheathless TFL lithotripsy. However, these associations are hypothesis generating, and causal inference cannot be established within the present study design. Nevertheless, impaired endoscopic visibility may necessitate frequent pauses to clear the view and re‐target the stone, likely contributing to the observed difference in laser‐use dynamics.

There are several plausible explanations for the surgeon reported reduced visibility in the HP group.^15^ Impaired endoscopic visibility due to increased turbulence, ‘snow‐storm’ effect, is a known association of high‐power (high‐frequency) dusting.^12^ Moreover, HP lithotripsy is prone to sparks from plasma formation,^18^ and stone ‘burning’ with carbonisation of the stone surface.^19^ Taken together, these effects may explain the reduced visibility when using HP TFL.

In the RCT, HP settings yielded faster stone ablation speed (mm^3^/s), yet this did not translate into improved procedural efficiency in terms of operative time or LOT.^15^ Meanwhile, LP yielded significantly superior SFR (Grade A: 63% and Grade B: 77%) compared with HP (Grade A: 44% and Grade B: 56%). In the current analysis, LP's superior workflow continuity (higher ODC) and better visualisation offer a plausible explanation for the higher SFR and comparable procedural efficiency observed with LP, despite its lower nominal power. HP's impaired endoscopic visibility and frequent start‐stop pattern may risk leaving small fragments obscured by dust or displaced by turbulence, undermining complete stone dusting. Thus, increased ablation speed without sustained visibility and controlled stone‐targeting does not necessarily translate into greater procedural efficiency or clinical outcomes.

Studies commonly report laser‐on time, energy delivered and operative time, but few characterise the temporal structure of laser activation. Earlier work of Aldoukhi et al. introduced the concept of ODC and described pedal activation patterns with Ho:YAG in a non‐randomised setting.^20^, ^21^ Yet, for TFL specifically, detailed intraoperative logs paired with clinical endpoints have been lacking. The current study extends this framework by providing the entire temporal structure of laser activation across all cases, linking it to operative time, endoscopic visibility and SFR within a randomised comparison of LP and HP TFL lithotripsy.

Importantly, all URS procedures in the RCT were performed without UAS. Although routine UAS use has not shown superiority over sheathless URS for clinical endpoints,^22^, ^23^ UAS utilisation can improve outflow and thereby help preserve endoscopic visibility.^24^ This is facilitated by the advent of flexible and navigable ureteral suction sheaths (FANS), which can deflect in tandem with the endoscope. Theoretically, FANS can help preserve endoscopic visibility during HP lithotripsy, and consequently, the observed divergence in activation patterns between LP and HP in the current study may be reduced. Nonetheless, a recent retrospective comparative study on FANS versus sheathless URS revealed similar 1‐month SFR: 91.8% and 90.5% for FANS and sheathless approach respectively.^25^ However, given the recency of FANS, the evidence base is sparse and future trials evaluating power settings in the context of FANS are warranted.

Understanding laser‐use dynamics provides a complementary dimension to interpreting laser performance. Metrics such as activation frequency, activation duration, pause duration and operator duty cycle capture how laser energy is applied in real time and may help reconcile why greater ablation speed does not necessarily translate into shorter procedures or higher SFR. Our findings suggest that *how* the laser is used, its activation pattern and continuity, may be as important as the intrinsic ablation rate of the power setting itself. LP settings promote more continuous lasing and may simplify workflow. In contrast, HP may require deliberate pause–reposition–retarget cycles to maintain precision in a sheathless environment.

The current study has certain limitations. The analysis was observational within a randomised framework and not powered specifically for all secondary endpoints. Nonetheless, observed differences were large and consistent. Moreover, the secondary analysis nature of this study introduces a risk of type 1 errors; to mitigate this, we report only multiplicity adjusted *p* values (Holm–Bonferroni).

As all procedures were performed without UAS, this reduces external validity beyond sheathless URS. Moreover, the HP group was restricted to 16–18 W for thermal safety. Although higher outputs are technically achievable with modern laser systems, power settings ≥20 W are generally discouraged during sheathless URS due to concerns regarding intrarenal temperatures.^11^ While we acknowledge that some authors may classify this range as ‘moderate power’, even higher power would likely yield larger differences between the groups in terms of laser activation patterns in a sheathless environment. Another limitation is that endoscopic visibility was assessed using surgeon‐reported Likert scores collected in the parent RCT. While these ratings were obtained prospectively and independently of the present analysis, they remain subjective and no objective intraoperative measure of visual clarity was available. Accordingly, associations between visibility and laser use dynamics should be interpreted as exploratory and non‐causal. Moreover, thermal safety‐driven adaptations and individual operator preference may influence laser activation behavior and cannot be fully excluded as potential confounders. However, procedures were performed by a large group of surgeons (11 residents and five consultants), which may mitigate the influence of any single training paradigm or individual preference on the observed activation patterns. To this end, HP and LP TFL lithotripsy yields distinctly different laser use dynamics during sheathless URS. These differences may have direct implications for operative workflow and the completeness of dusting. Accordingly, power selection should be guided not only by ablation speed but also by the ability to maintain steady laser activation under a clear visual field, which may be particularly relevant in sheathless URS.

## 7 CONCLUSION

Low‐ and high‐power TFL lithotripsy exhibit distinctly different laser‐use dynamics during sheathless URS. Low‐power supports sustained, continuous lasing, whereas high‐power yields intermittent, stop–start activation. Coupled with the RCT outcomes of higher SFR and fewer postoperative complications in favour of LP lithotripsy, these findings suggest that sustained lasing under clear visualisation may be an important determinant of TFL performance. Selecting power settings that support sustained laser activation may be more effective than simply escalating wattage.

## AUTHOR CONTRIBUTIONS

**Mathias S. Æsøy:** Conceptualization; methodology; investigation; resources; data curation; formal analysis; visualization; writing—original draft; writing—review and editing. **Christian Beisland:** Methodology; resources; writing—review and editing; supervision. **Øyvind Ulvik:** Conceptualization; methodology; investigation; resources; data curation; writing—original draft; writing—review and editing; supervision; project administration.

## CONFLICT OF INTEREST STATEMENT

Mathias S. Æsøy and Øyvind Ulvik have received honoraria for lectures and courses sponsored by Olympus. Olympus has not been involved in any part of the present study. The other authors have nothing to disclose.
